# Hematological malignancies in the Northwest Ethiopia

**DOI:** 10.1371/journal.pone.0260639

**Published:** 2021-12-01

**Authors:** Bamlaku Enawgaw, Melak Aynalem, Mulugeta Melku, Fikir Asrie, Molla Abebe, Aregawi Yalew, Tiruzer Bekele, Nebiyu Mesfin, Mulugeta Ayalew, Elias Shiferaw

**Affiliations:** 1 University of Gondar, College of Medicine and Health Science, School of Biomedical and Laboratory Sciences, Department of Hematology and Immunohematology, Gondar, Ethiopia; 2 University of Gondar, College of Medicine and Health Science, School of Medicine, Department of Pathology, Gondar, Ethiopia; 3 University of Gondar, College of Medicine and Health Science, School of Medicine, Department of Internal Medicine, Gondar, Ethiopia; 4 University of Gondar, College of Medicine and Health Science, School of Medicine, Unit of Pediatric Hematology Oncology, Gondar, Ethiopia; Qatar University, QATAR

## Abstract

**Background:**

The effect of malignant diseases is increasing globally, particularly in developing countries as shown by recent cancer statistics from the world health organization reports. It is anticipated that with an increase in life expectancy consequent upon the improved standard of living and increasing urbanization, the burden of hematological malignancies in sub-Saharan Africa particularly in Ethiopia is likely to increase recently. Therefore, this study was aimed to determine the incidence and trend of hematological malignancy in Northwest Ethiopia.

**Methods:**

A facility-based retrospective study was conducted from 2015 to 2019 at the University of Gondar and Bahir-Dar Felegehiwot comprehensive specialized hospitals. Hematological malignancy data were collected by using a data collection sheet that was consisted of patients’ socio-demography, clinical, and laboratory data. Then, data were entered into Epi-info 3.5.1 and exported to SPSS version 20 for analysis. Skewness and kurtosis were used to check data distribution. Descriptive statistics were summarized as percentages, means, and standard deviations of background variables, and the trend were analyzed.

**Results:**

In this study, a total of 1,342 study participants were included. The mean age of study participants was 41.49 ± 16.3 years with a range of 1 to 92 years. About 58.3%, 52.2%, and 80% of the cases were observed among males, 18–45 age group, and urban residences, respectively. Of the total cases, 92.9% and 7.1% were lymphoma and leukemia, respectively. On the other hand, from lymphoma cases, 72.3% and 27.7% were HL and NHL, respectively while from leukemic cases, 61.1%, 23.2, 6.3%, 4.2%, and 5.3% were CLL, ALL, CML, AML, and other HM types, respectively. In this study, there was no trend.

**Conclusion:**

We concluded that lymphoma was the dominant type of hematological malignancy observed in northwest Ethiopia. The study indicated that the majority of cases were observed among male, urban residents, and adult populations aged 18–45 years. Therefore, special focus should be given to the highly affected population. Further, a prospective cohort study should be conducted for a better understanding of the prevalence and associated factors to it.

## Background

Hematologic malignancies (HM) are a group of neoplasms that comprises a collection of heterogeneous conditions, all originating and transformed from cells of the bone marrow and the lymphatic system. These malignancies are clonal disorders that result in autonomous cell proliferation, suppressing normal hemopoietin, and also infiltrating tissues and organs [1, 2].

Traditionally, HM was regarded as leukemia, lymphomas, and myeloma. But, the 2008 world health organization (WHO) classification system recognized over 60 different clinical and pathological disease subtypes [3]. Besides, HM can also be classified as acute that can be rapidly fatal, if untreated; many others are insidiously progressive and become chronic, leading to eventual death. The acute types are often malignancies emanating from precursor cells while the insidious ones are often malignancies arising from relatively or fully matured cells [1–3].

Leukemia is a rare leukocyte and its precursor disease that can cause higher mortality than many of the acute communicable diseases because of its fatal character [4]. Based on cell type, differentiation, morphology, cytochemical characteristics, and immune-phenotyping, leukemia can be classified as acute or chronic [5, 6]. Acute leukemia comprises acute lymphoblastic leukemia (ALL) and acute myeloblastic leukemia (AML), while chronic leukemia includes chronic lymphocytic leukemia (CLL) and chronic myelocytic leukemia (CML) [2, 7, 8].

Lymphoma is a lymphoid malignancy that involves lymph nodes and/or other extramedullary sites. They are broadly classified as being Hodgkin lymphoma (HL) and non-Hodgkin lymphoma (NHL) and both are treated with very different protocols. The NHL may be immature lymphoblastic, although the vast majorities have a mature B, T, or natural killer cell phenotype although 25% of childhood NHL derives from precursor (immature) lymphoblasts. Extramedullary myeloid proliferations are unusual and most commonly seen in the setting of AML, in which case it represents a myeloid sarcoma (extramedullary accumulation of myeloid blasts). Histiocytic and dendritic cell neoplasms are generally rare except for Langerhans cell histiocytosis (an entity whose neoplastic nature is controversial), which is seen relatively more common, especially in children [5, 6].

The rising global incidence of malignant diseases as documented in recent reports of the WHO is an issue of serious concern, particularly in developing countries where the increase is occurring at a faster rate. In 1985, there were an estimated 7.62 million new cancer cases, with 3.66 million (48%) in the developed countries and 3.96 million (52%) in the developing countries [9]. But in 1990 this number rose to 8.1 million new cases [10]. In the same year 1990, it was estimated that 5.2 million cancer deaths occurred, with about 55% occurring in developing countries. With increasing industrialization and westernization of dietary and other socio-behavioral attitudes in most developing countries, it is estimated that the burden of cancer in these countries will increase to epidemic proportions in the 21st century. Worldwide, over 250,000 people are diagnosed with leukemia each year, accounting for 2.5% of all cancers [10].

Hematologic malignancies have emerged as a major cause of morbidity and mortality in sub-Saharan Africa. The NHL, HL, leukemia, and multiple myeloma (MM) together accounted for 8.7% of incident cancer diagnoses and 9.9% of cancer deaths in 2016, with NHL being the sixth most common cancer in the region [11]. Hematologic malignancies have become the cause of morbidity and mortality in developing countries. However, in Ethiopia, there is no significant information regarding this condition and it was felt to be important to fill in the gaps in knowledge which might inform future healthcare initiatives. Therefore, this study was designed to determine types and trends of HM at the two major referral hospitals in the North East region.

## Materials and methods

### Study design, area and period

This retrospective study was conducted at the University of Gondar comprehensive specialized hospital (UoG-CSH) and Bahir-Dar Felegehiwot comprehensive specialized hospital from 2015 to 2019. The UoG-CSH is found in Gondar town which is 737 km away from Addis Ababa, the capital city of Ethiopia. The hospital is providing different medical services to more than seven million people in the region and people of the neighboring region. It provides adult and pediatric hematology and oncology services to the region. Its pediatric hematology-oncology center is the only of its kind in the region. Basic imaging and pathologic services are available. Also, Bahir-Dar Felegehiwot comprehensive specialized hospital is found in Bahir-Dar town which is 492 km away from Addis Ababa, the capital city of Ethiopia. The hospital is designed as a referral hospital for the Amhara region and the region has more than 30 million people.

### Study population and sampling procedures

The study population consisting of all patients diagnosed with hematological cancer at the participating centers was taken as the study population for the magnitude and trend of HM. This study includes all HM patients having minimum information (sociodemographic data, and laboratory results) and diagnosed from 2015 to 2019.

### Data collection procedure

Hematological malignancies data that were diagnosed by using the bone marrow aspiration and biopsy at the UoG-CSH and Bahir-Dar Felegehiwot comprehensive specialized hospital were collected. All cases of HM data have been retrieved and reviewed carefully. For those cases, the patient’s full data on the logbook that includes the name, gender, age, residence, date of diagnosis, and type of HM were collected with an information sheet that was being prepared for this study. While any HM patient data which were not fully documented were excluded from the study. To ensure the quality of data, training was given to data collectors and supervisors before the beginning of data collection.

### Data analysis

The data were entered into EPI-Info version 3.5.3 and data completeness was checked, then the data were transported into SPSS version 20 for data analysis. Skewness and kurtosis were used to check data distribution. Continuous numeric variables were expressed as the mean ± standard deviation for the normally distributed data but the skewed data were expressed in median and IQR. Categorical variables were described by using the frequency numbers and percentages. Then, descriptive statistics were summarized as percentages, means, and standard deviations. Presented with figures and tables. Further, the trend of HM was computed for both study areas.

### Ethical approval and consent to participate

In this retrospective study, obtaining informed consent was not possible. So, we were granted a waiver of consent by the Ethical Review Committee, and patient records/information was anonymized and de-identified before analysis. The study protocol was reviewed by the Institutional Review Boards of the University of Gondar (Ethical Clearance letter reference number: O/V/P/RCS/05/378/2017). To ensure confidentiality of data code was used. Any unauthorized persons were no access to the collected data. An information sheet containing names was kept locked.

## Results

### Sociodemographic of study characteristics

A total of 1,342 study participants with different types of HM was collected. The mean age of study participants was 41.49 ±16.3, ranging from 1–92 years. Out of the total study participants, 58.3% were male and 92% were adults. The majority of the participants (80%) was from the urban residence (Table 1).

**Table 1:** Sociodemographic characteristics of study participants at UoG-CSH and Bahir-Dar Felegehiwot comprehensive specialized hospital: Northwest Ethiopia.

Variables	Categories	Frequency	Percentages
**Sex**	Male	782	58.3%
	Female	560	41.7%
**Age in years**	<18	106	7.9%
	18–45	701	52.2%
	46–65	447	33.3%
	>65	88	6.6%
**Residence**	Urban	1073	80.0%
	Rural	269	20.0%

### Types of hematological malignancy

**Fig 1:**
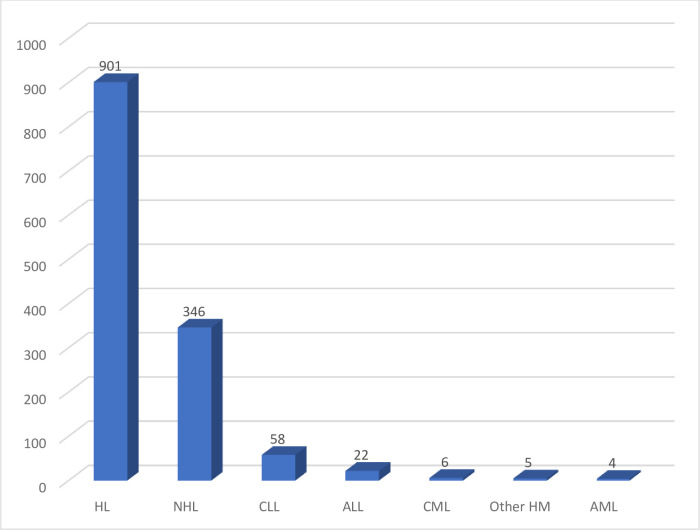
Hematological malignancy among study participants at UoG-CSH and Bahir-Dar Felegehiwot comprehensive specialized hospital: Northwest Ethiopia.

Of the total study participants, 92.9% (1247/1342) were diagnosed with lymphoma and 7.1% (95/1342) with leukemia. The median age of the lymphoma and leukemia diagnosed study participants was 41 (IQR = 10) and 54 (IQR = 26) respectively. Further, from lymphoma patients, 72.3% (901/1247) and 27.7% (346/1247) of them were HL and NHL respectively. Among leukemic patients 61.1% (58/95), 23.2% (22/95), 6.3% (6/95), 4.2% (4/95), and 5.3% (5/95) developed CLL, ALL, CML, AML, and other HM, respectively (Fig 1).

#### Gender, residence, and age ratio

In the current study, among NHL, HL, and Leukemic cases the male study participants account for 57.6% (718/1247), 66.18% (229/346), 54.27% (489/901), 67.37% (64/95) respectively. On the other hand, HM was common in urban residences than in rural ones. Of the HM cases, about 80% were from urban residences. Similarly, from lymphoma and leukemia cases 80.2% and 76.8% were from urban residences. From NHL, HL, ALL, CLL, AML, CML, and other HM types the urban resident individuals accounts 84.9%, 67.9%, 72.7%, 75.9%, 75%, 83.3%, and 100% of all cases. Based on age classification, of the lymphoma cases, the 19–45 age group accounts for 54% and 57% of the HL and NHL cases respectively. Also from leukemic cases ALL, CLL, and AML were dominant in the age group of 19–45, 46–65, and >65 years (Table 2).

**Table 2:** Classification of hematological abnormality of study participants at UoG-CSH and Bahir-Dar Felegehiwot comprehensive specialized Hospital: Northwest Ethiopia.

Types of HM	Gender		Residence			Age group in years		
	Male	Female	Urban	Rural	<18	19–45	46–65	>65
**Lymphoma(n = 1247)**	718 (57.6%)	529 (42.4%)	1000(80.2%)	247(19.8%)	95 (7.6%)	674 (54%)	408 (32.7%)	70 (5.6%)
**HL (n = 901)**	489 (54.3%)	412 (45.7%)	765 (84.9%)	136(15.1%)	52 (5.8%)	521 (57%)	279 (31%)	49 (5.4%)
**NHL (n = 346)**	229 (66.2%)	117 (33.8%)	235 (67.9%)	111(32.1%)	43 (12.4%)	153 (44.2)	129 (37.3%)	21 (6.1%)
**Leukemia (n = 95)**	64 (67.4%)	31 (32.6%)	73 (76.8%)	22(23.2%)	11(11.6%)	27(28.4%)	39(41.1%)	18 (18.9)
**ALL (n = 22)**	20 (90.9%)	2 (9.1%)	16 (72.7%)	6 (27.3%)	9 (40.9%)	10 (45.5%)	3(13.6%)	0
**CLL (n = 58)**	38 (65.5%)	20 (34.5%	44 (75.9%)	14 (24.1%)	2 (3.4%)	14(24.1%)	26 (44.8%)	16 (27.6%)
**AML (n = 4)**	3 (75%)	1 (25%)	3 (75%)	1 (25%)	0	1(25%)	1 (25%)	2 (50%)
**CML (n = 6)**	0	6 (100%)	5 (83.3%)	1 (16.7%)	0	0	6(100%)	0
**Other HM (n = 5)**	3 (60%)	2 (40%)	5 (100%)	0	0	2(40%)	3(60%)	0

### Trends of hematological malignancy

**Fig 2:**
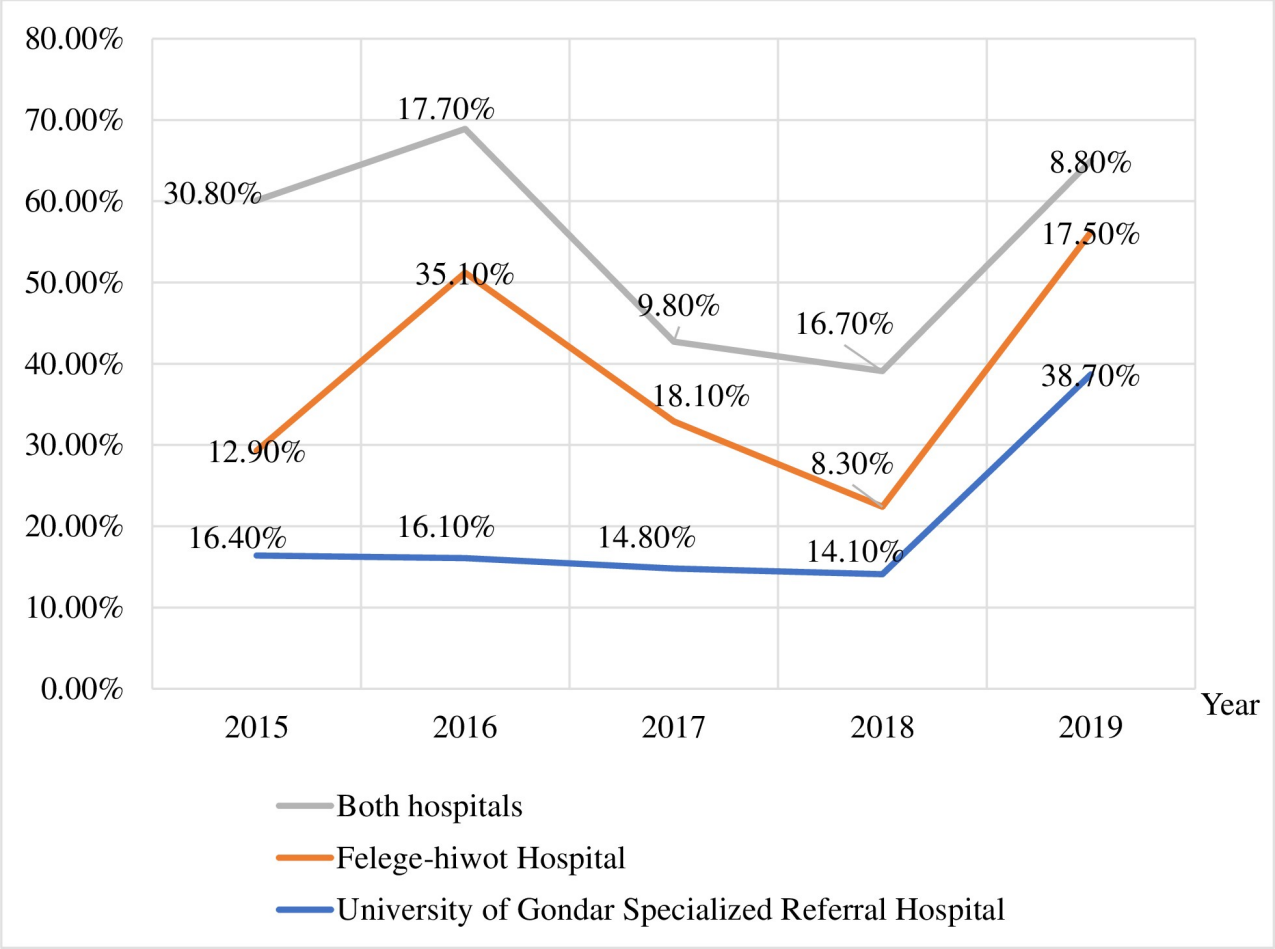
Year trend of hematological malignancy among study participants: Northwest Ethiopia.

A total of 1,342 HM was diagnosed from 2015 to 2019. The magnitude of HM at Bahir-Dar Felegehiwot comprehensive specialized hospital was 12.92% (134/1,037), 35.10% (364/1,037), 18.22% (189/1,037), 8.29% (86/1,037), and 17.45% (181/1,037) and at the UoG-CSH was 16.4% (50/305), 16.1% (49/305), 14.8% (45/305), 14.1% (43/305), and 38.7% (118/305), in the year 2015 to 2019, respectively. The overall magnitude of HM at both hospitals was 30.8% (413/1,342), 17.7% (238/1,342), 9.8% (132/1,342), 16.7% (224/1,342), and 8.8% (118/1,342) from 2015 to 2019, respectively (Fig 2).

## Discussion

Cancer is a common public health problem. Globally, HM represents the fifth most frequently diagnosed cancer type [12]. The incidence of cancer is increasing and has now become a global burden, and it is the second leading cause of death in the United States [12, 13]. In the sub-Saharan region of Africa, the incidence of HM is escalating in large part because of infectious disease epidemics like the human immunodeficiency virus as well as population growth and aging [14]. Also, the incidence increased particularly in developing countries including in Ethiopia [15, 16]. Therefore, this study aimed to determine the incidence and trend of HM in Northwest Ethiopia.

In this study, of the HM cases, the magnitude of lymphoma among HM was 92.9% (95% CI: 91.99, 93.81), and the rest 7.1% (95% CI: 6.23, 7.97) were leukemic. This study showed that nine participants out of ten had developed different types of lymphoma. It indicates that the majority of study participants were lymphoma patients. The incidence of lymphoma in the current study was higher than a global report in 2020 the incidence of lymphomas, leukemia, and MM was 48%, 34%, and 18% [17] Similarly, it is higher than a study conducted in the USA, Canada, Chile, Morocco, South Africa, East Africa, Ethiopia, Addis Ababa, and Gondar showed that the prevalence of Lymphoma among HM patients was 63.33%, 40%, 56.5%, 29%, 68.1%, 44%, 49.7%, 53.7%, and 18.5% [16, 18–25]. The discrepancy might be related to the current study majorly including the adult population (91%) and these populations are known to be more affected by lymphoma than leukemia. In addition to this, the majority of the current study specimens were taken from neck, leg, hand, and lymph nodes (11.0%, 22.4%, and 15.4% respectively). Only 0.6% of specimens were taken from bone marrow.

The current study also revealed that among Lymphoma patients the prevalence of HL and NHL was 72.3% (95% CI: 71.8, 72.8) and 27.7% (95% CI: 27.2, 28.2) respectively. The prevalence of NHL was lower than a global report which showed 54.5% of NHL among HM [19]. Similarly, it is lower than a study conducted in USA, Canada, East Africa, Ethiopia, and Addis Ababa, 90.1%, 83%, 83.6%, 82.7%, and 97.1% [16, 17, 19, 24, 26]. The discrepancy might be related to variation in the availability of diagnostic tools and experts, study population, study area, study design, and socioeconomic factors.

In the current study, the prevalence of Leukemia among HM patients was 7.1% (95% CI: 3.82, 10.38). Despite the confounding factor of where the diagnostic specimens were taken, leukemia appears to be by far the least type of HM found among study participants. The current study result is lower than a global report in 2020 the prevalence of leukemia was 34% [19]. Similarly, it is lower than a study conducted in the USA, Canada, Chile, Morocco, South Africa, East Africa, Ethiopia, Addis Ababa, and Gondar showed that the prevalence of Leukemia among HM patients was 33.33%, 27%, 19.6%, 22.2%, 47.4%, 50.1%, 44.3%, and 30.2% respectively[16–18, 21–24, 26]. Also, in this study, CLL (61.1%) was the most predominant leukemia type followed by ALL (23.2%). This result is similar to a global and Canada report that showed CLL was the most predominant type of HM followed by ALL[17, 19]. In contrast, a report in East Africa showed that CML was the predominant type of HM [22]. The result variation may be related to the current study was majorly (92.1%) included the adult population. But the leukemia malignancy is higher in children [25]. For those patients having HM the available treatment options in the study settings are conventional chemotherapy regimens, intrathecal therapies and at times targeted therapies like tyrosine kinase inhibitors, monoclonal antibodies mainly Rituximab-based therapies. The availability of chemotherapeutic drugs has improved much over the last few years. Treatment is usually complicated by the low level of supportive care at the treating centers, lack of sustainable blood and blood product supply, shortage of antibacterial and antifungal.

In the current study, males were dominantly affected by both lymphoma (57.6%) and Leukemia (67.4%). Hence, male study participants were more exposed to different HM than females. This is due to male study participants being more exposed to different risk factors that induce HM than females [27]. This study is in line with a study conducted on HM disorders [2]. Besides, the urban residence study participants (80% of the total HM) were more affected than a rural areas. However, in the Ethiopian and Amhara region context, about 79.3%, and 87.73% of the total population are live in rural residents. Similarly, from lymphoma and leukemia cases about 80.2% and 76.8% were from urban residences respectively. This indicates that four individuals out of five cases were living in urban residences. The reason for the high result might be that living in an urban area may expose the individuals to different pollutants (e.g. chemicals, light, and noise), novel types of food, and new infections that can cause HM [28]. Further, based on age classification, being 19–45 years old was more affected by lymphoma (54%) and leukemia (45.5%). This showed that the NHL was commonly found in the age group of 19–45 years. The current study showed that being an adult is mostly affected by lymphoma malignancy which is similar to different studies [2]. Also, this result showed that the younger age study participants were dominantly affected by lymphoma than leukemia.

The trend of HM in the current study, from 2015 to 2019 in the Bahir-Dar Felegehiwot comprehensive specialized hospital and UoG-CSH was not observed. Similarly, in both sites from 2015–2019 the magnitude of HM was 30.8%, 17.7%, 9.8%, 16.7%, and 8.8% respectively. This illustrated that there was no trend in these sites. In contrast to the current study, a report in the USA showed that the overall cancer incidence rate in men declined by approximately 2% per year [13]. Similarly, age-adjusted rates for new leukemia cases have been stable over 2008–2017. But, age-adjusted rates for new NHL cases have been falling on average 0.8% each year over 2008–2017[17]. Also, the number of cases of all cancers has been increasing steadily over the years, from 1998 to 2018. Compared to all cancers, leukemia cases have been increasing much faster, from 1998 to 2018, with an abrupt increase between the years 2006 and 2007 [29].

### Strength and limitations

This study has some strengths and limitations. As of its strength, the study includes multiple center data and includes extensive data collection. But as a limitation, due to the limited availability of information from the registration book, the authors were not able to further identify the exact causes of the correlations between the magnitude of HM and the associate factors, the mortality, the effectiveness, and availability of treatment.

## Conclusion and recommendation

Hematological malignancy more prominently lymphoma, poses a substantial public health problem in the Amhara region. From lymphoma and leukemia cases NHL and CLL were dominant. Study participants living in urban areas and males were highly affected with HM than rural and females. Therefore, the policymaker should give special concern for the male and the population living in urban residences. Also having accurate diagnosis, treatment, and prevention of HM in developing countries like Ethiopia is very critical. Data collection and accurate analysis will aid better understanding of HM in the study area and allow better projecting and planning for future strategies to respond to the growing burden of hematological cancer diseases.

## List of abbreviations

ALL: acute lymphocytic leukemia
AML: acute myeloid leukemia
CML: chronic myeloid leukemia
CLL: chronic lymphocytic leukemia
HL: Hodgkin’s lymphoma
HM: hematological malignancies
MM: Multiple Myeloma
NHL: Non-Hodgkin’s lymphoma
UoG-CSH: University of Gondar Comprhensive and Specialized Hospital
WHO: World Health Organization
